# “Bitcoin now”: temporal discounting in Bitcoin holders

**DOI:** 10.3389/fnins.2023.1205814

**Published:** 2023-07-13

**Authors:** Mohamad El Haj, Ahmed A. Moustafa

**Affiliations:** ^1^Nantes Université, Univ Angers, Laboratoire de Psychologie des Pays de la Loire (LPPL – EA 4638), Nantes, France; ^2^CHU Nantes, Clinical Gerontology Department, Nantes, France; ^3^Institut Universitaire de France, Paris, France; ^4^School of Psychology, Faculty of Society and Design, Bond University, Gold Coast, QLD, Australia; ^5^Department of Human Anatomy and Physiology, The Faculty of Health Sciences, University of Johannesburg, Johannesburg, South Africa

**Keywords:** Bitcoin, cryptocurrency, decision making, temporal discounting, behavioral economy

## Abstract

**Introduction:**

Cryptocurrency investment and trading are rapidly growing activities due to the development of applications and platforms that offer fast, continuous, and easy entry into the cryptocurrency world. To understand decision making in cryptocurrency holders, we assessed temporal discounting, that is, whether Bitcoin holders disregard rewards if they are distant in time and overvalue rewards if they are more immediate. Further, we compared performance between short-term investors (i.e., day-traders) vs. long-term investors.

**Methods:**

Using an online survey, we invited 144 Bitcoin holders to answer temporal discounting questionnaires dealing with money (“Which do you prefer, that you get right now 20 USD in cash or 100 USD in a month?”) and Bitcoin (“Which do you prefer, that you get right now 0.1 or 1 Bitcoin in a month?”).

**Results:**

Analysis demonstrated no significant differences between temporal discounting for money and Bitcoin. However, and critically, higher temporal discounting for both money and Bitcoin was observed in short-term investors compared with long-term investors. In a similar vein, significant positive correlations were observed between day trading and temporal discounting for both money and Bitcoin.

**Discussion:**

These findings demonstrate how Bitcoin holders with short-term time horizons tend to prioritize immediate rewards over larger but delayed rewards. Future research can assess the neural basis of temporal discounting for cryptocurrencies.

## 1. Introduction

Cryptocurrency can be defined as a digital money or a medium of exchange wherein ownership and transfer information is recorded and kept in a digital ledger in which ownership is protected using blockchains (Oksanen et al., 2022). Blockchains are distributed nodes of ledgers where each ledger is linked together in a peer-to-peer network. Based on blockchain technology, the first cryptocurrency (i.e., Bitcoin) was created in 2009, followed by alternative coin (i.e., altcoins) in 2011. Although the value of cryptocurrency is much more volatile than classical investment tools, Bitcoin is becoming a widely accepted exchange and investment tool. Because cryptocurrency trading can be risky, a growing body of literature is focusing on whether cryptocurrency trading can be associated with abnormal behaviors and decision making (Mills and Nower, 2019; Delfabbro et al., 2021; Sonkurt and Altinoz, 2021; Oksanen et al., 2022). In line with this literature, the present paper investigates the relationship between cryptocurrency trading and temporal discounting.

Temporal discounting refers to the tendency to disregard rewards if they are distant in time and overvalue rewards if they are more immediate (Chapman and Elstein, 1995; Green and Myerson, 2004; Frederick, 2006). Typical assessments of temporal discounting involve monetary incentive questionnaires on which participants are invited to indicate whether they prefer immediate amount of money over delayed, but larger, amount of money, with variation amount of the immediate reward but a fixed amount of the future reward (e.g., “Which do you prefer, you get 10 USD right now or 100 USD in a month?” and “Which do you prefer, you get 20 USD right now or 100 USD in a month?”) (El Haj et al., 2020, 2022, 2023; El Haj and Moustafa, 2023a,b). Although some degree of temporal discounting may be economically advantageous (Frederick et al., 2002), excessive temporal discounting can be associated with shortsighted and risky behaviors such as gambling (Holt et al., 2003), substance dependence (Bickel et al., 2011), and risky sexual behaviors (Chesson et al., 2006). Excessive temporal discounting has been even suggested as a candidate behavioral marker for maladaptive behaviors such as overeating (Amlung et al., 2016) and addiction (Kwako et al., 2018).

In the current study, we investigated whether cryptocurrency holding can be associated with temporal discounting (i.e., preference for immediate amount of money, over delayed, but larger amount of money). Critically, we investigated whether temporal discounting may be excessively observed for Bitcoin. Specifically, we examined whether cryptocurrency holders exhibit a strong preference for an immediate amount of Bitcoin over a larger amount of Bitcoin that is delayed. This expectation was based on research demonstrating how riskier and volatile financial instrument investments are associated with riskier behaviors. Our expectation was also based on research suggesting how cryptocurrencies can be related to high-risk stocks and options (Mills and Nower, 2019). Although cryptocurrencies, especially stable coins (i.e., cryptocurrencies pegged by other stable assets), are widely accepted as a relatively stable investment instrument, cryptocurrency holding encompasses volatile and risky investment tools such as margins, options, and future trading. These cryptocurrency investment tools may activate problem gambling behavior such as the “rush” behavior whereby users may trade highly volatile stocks for the highs and lows of the volatility experience *per se* rather than for strategic investment (Arthur et al., 2016b; Youn et al., 2016; Grall-Bronnec et al., 2017). In other words, cryptocurrency holding and trading may involve risky decisions. These decisions may mirror high levels of temporal discounting by which holders would prefer the immediate over later, although better, rewards. This tendency can be compared with the model of Arthur et al. (2016b) who described a spectrum between gambling and investment with, in the middle of the spectrum, speculative traders who tend to demonstrate behaviors similar to gamblers. Support for the relationship between excessive trading and gambling can be found in research on pathological trading. This research has shown that pathological trading affects both mental and physical health by increasing depression, anxiety disorders, and suicide attempts (Guglielmo et al., 2016; Johnson et al., 2023). Taken together, cryptocurrency trading may involve risky and speculative decisions. We thus expected high levels of temporal discounting in cryptocurrency holders, especially when the temporal discounting choice involves Bitcoin.

In our attempt to assess temporal discounting in cryptocurrency holders, we considered sociodemographic factors such as age, gender, and education. We considered age because risky gambling is believed to peak in the late teens or early 20s, and then declines throughout life (Mok and Hraba, 1991; Welte et al., 2001). Furthermore, research has demonstrated that female stock market investors tend to take less risky decisions and show less shortsighted trading compared to male ones, and that those who take the higher risky decisions are typically young and educated men (Jadlow and Mowen, 2010; Deck et al., 2014; Arthur et al., 2016a). In addition to sociodemographic factors, we considered a core trading strategy, that is, whether holders consider short vs. long-term investment. In trading in general, a distinction can be made between short vs. long-term trading. Short-term trading is associated with shortsighted and gambling-like decisions as well as with inconsistent, and even poor, returns for investors (Barber et al., 2009; Dorn et al., 2014; Gao and Lin, 2015; Arthur et al., 2016a,b). This can be especially true for cryptocurrency traders as gamblers who engage in short-term and risky decisions are more likely to report cryptocurrency trading (Mills and Nower, 2019). We thus reasoned that, compared to long-term holders, short-term holders tend to consider short-term time intervals and, thus, may be probably keen to more prefer immediate over long-term crypto rewards (i.e., to demonstrate increased temporal discounting).

To summarize, cryptocurrency investing is increasingly becoming a mainstream behavior. Despite the social and economic weight of cryptocurrency, relatively little research has assessed decision making in this market domain. However, research suggests that cryptocurrency trading can be associated with risky behaviors and decision making (Mills and Nower, 2019; Delfabbro et al., 2021; Sonkurt and Altinoz, 2021; Oksanen et al., 2022). To provide an in-depth assessment of decision making in cryptocurrency holders, we assessed whether holders may tend to demonstrate increased temporal discounting when reward involves Bitcoin. We expected that excessive temporal discounting would be observed to a greater extent when the reward involves Bitcoin rather than money, especially in short-term holders. These outcomes can mirror how cryptocurrency investment can activate short-term time-horizons decision in holders. In order to examine the time-horizons, we invited participants to answer two questions (1) whether they consider cryptocurrencies as a long- or short-term investment and (2) whether they use day trading (i.e., buying/selling Bitcoin within the same trading day such that all orders will be fulfilled before the market closes for the day).

## 2. Materials and methods

### 2.1. Participants

The online-based survey study included 144 participants (28 women, 116 men, *M* age = 26.18 years, SD = 9.74, *M* formal education = 14.78 years, SD = 4.66). Online recruitment was implemented through top cryptocurrencies forums: Reddit, Steemit, Bitcointalk, and Bitcoin.com. Reddit is a basic online community of millions of users engaging in the discussion of general interests. Although Steemit, Bitcointalk, and Bitcoin.com include less users compared to Reddit, these forums are dedicated for people interested in trading, technical details, and the development of cryptocurrencies. Prior to taking the survey, participants were informed that the aim of the study was to assess their perspectives of trading. As part of the consent form, participants were informed that their responses would be anonymously analyzed for research purposes. Regarding the inclusion criteria, we included only participants above 18 years old as well as those who hold Bitcoin in their cryptocurrency wallets (i.e., software/hardware storing and managing Bitcoins transactions). Participants were not able to take the survey prior to providing their consent and disclosing their age (i.e., to ensure that they were major) and holding Bitcoins. We excluded nine participants from the original sample (*n* = 153) who completed the entire survey very fast (i.e., in less than 1 min compared to the mean completion time of 3 min). The final sample (*n* = 144) included only participants who responded to the entire survey.

While designing the study, we calculated *a priori* the sample size using G*Power (Faul et al., 2007). Calculation was conducted for within-subjects measurements two tailed *t*-tests as our experimental design involved two repeated conditions (i.e., temporal discounting for money vs. Bitcoin). The sample size calculation was also based on 95% power, an estimated probability of making Type I error as 0.05, and a medium effect size of 0.5 (Cohen, 1992). While the calculation has suggested a total sample size of 54 participants, we recruited a larger sample during the study implementation (i.e., from February to May 2022). The final sample size (*n* = 144) thus provides fair statistical power regarding the study design.

### 2.2. Procedures

The survey was developed online using SurveyMonkey. The survey opened with sociodemographic data (i.e., age, gender, and educational level) and by asking participants if they hold Bitcoin or not. Afterward, participants answered a question about their short vs. long-term holding horizon (i.e., “are you holding Bitcoin for short or long term?” reply = yes or no). Participants also answered the following question (i.e., “do you trade Bitcoin daily?”) using a five points-scale (0 = never, 1 = rarely, 2 = sometimes, 3 = frequently, 4 = always). Afterward, participants answered to questionnaires: one of them was focused on answering about money, and the other about Bitcoin. The order of the two questionnaires were counterbalanced across participants.

Temporal discounting questionnaires were inspired by a task proposed by Boyle et al. (2012, 2013) who developed an easy-to-administrate task consisting of binary questions. Furthermore, Boyle et al. (2012, 2013) found high correlations between their test and standard tests of temporal discounting, which indicates high reliability (Kirby and Maraković, 1996; Critchfield and Kollins, 2001). On our “money temporal discounting” questionnaire, participants were invited to answer 10 questions, each of which involves choosing between two options, either an immediate but smaller amount of money or a delayed but larger amount of money (e.g., “Which do you prefer, you get 100 USD right now or 1000 USD in a month?” and “Which do you prefer, you get 200 USD right now or 1000 USD in a month?”). The delayed amount of money was fixed at 1,000 USD. We however fixed the delay, in line with the procedures of Boyle et al. (2012, 2013), at 1 month. However, we variated and counterbalanced the immediate amounts across the ten questions as provided in the Appendix. The same procedures were applied for the “Bitcoin temporal discounting” questionnaire. However, the 10 questions involved choosing between two options, either an immediate but smaller amount of Bitcoin or a delayed but larger amount of Bitcoin (e.g., “Which do you prefer, you get 0.1 Bitcoin right now or 1 Bitcoin in a month?” and “Which do you prefer, you get 0.2 Bitcoin right now or 1 Bitcoin in a month?”). The delayed amount of money was fixed at 1 Bitcoin and the delay was fixed at 1 month, however, the immediate amounts variated, while counterbalanced, across the ten questions (i.e., 0.1, 0.2, 0.3, 0.4, 0.5, 0.6, 0.7, 0.8, 0.9, and 1 Bitcoin).

The temporal discounting score was the shortsighted choice ratio, namely, the proportion of answers in which participants chose the smaller immediate reward divided by the total number of answers. Therefore, high scores indicated very shortsighted decisions. Note that, unlike other scores of temporal discounting such as the hyperbolic discount rate or parameters derived from quasi-hyperbolic models, the shortsighted choice ratio, as used in our study, is a simple indicator that requires no assumptions about the shape of the discount function (Kayser et al., 2012; Chiong et al., 2016). Furthermore, research has reported strong correlations between this simple indicator and the hyperbolic discount rate (Boettiger et al., 2007; Kayser et al., 2012; Lebreton et al., 2013).

### 2.3. Statistical analysis

To test our hypothesis (i.e., higher temporal discounting when the reward involves Bitcoin rather than money), we used paired *t*-tests to compare the shortsighted choice ratio between the “money temporal discounting” and “Bitcoin temporal discounting” questionnaires, after checking for normal distribution of data with using Shapiro–Wilk tests. We also carried-out Pearson correlations between temporal discounting and sociodemographic (i.e., age, gender, and education level) and trading variables (i.e., short- vs. long-term horizons, and day trading).

## 3. Results

### 3.1. No significant differences between temporal discounting for money and Bitcoin

**FIGURE 1:**
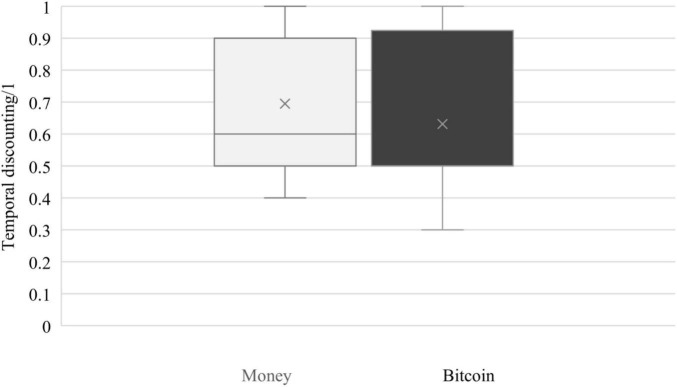
Temporal discounting for money and Bitcoin.

As shown in Figure 1, and contrary to our hypothesis, no significant differences were observed between temporal discounting for money (*M* = 0.61, SD = 0.27) and Bitcoin (*M* = 0.59, SD = 0.26), *t*(143) = 0.75, *p* = 0.455. We, however, compared the temporal discounting scores vs. midpoint scores (i.e., 0.5), in order to highlight whether these scores can be considered as high or low compared to the mean score. Analysis demonstrated that temporal discounting for money, *t*(143) = 4.75, *p* < 0.001, and Bitcoin, *t*(143) = 4.24, *p* < 0.001, was higher than the mean.

### 3.2. High temporal discounting in short-term investors

Analysis demonstrated higher temporal discounting for money in short-term (*M* = 0.77, SD = 0.23) than in long-term (*M* = 0.43, SD = 0.21) holders, *t*(142) = 9.15, *p* < 0.001. Analysis also demonstrated higher temporal discounting for Bitcoin in short-term (*M* = 0.76, SD = 0.18) than in long-term holders (*M* = 0.42, SD = 0.24), *t*(142) = 9.92, *p* < 0.001. Regarding within groups comparisons, analysis demonstrated no significant differences between temporal discounting for money and Bitcoin in short-term holders, *t*(74) = 0.32, *p* = 0.75, or in long-term holders, *t*(68) = 0.82, *p* = 0.41. Furthermore, no significant differences were observed regarding the distribution of short-terms (*n* = 75) vs. long-term holders (*n* = 69) in our sample [χ^2^(1, *N* = 144) = 0.25, *p* = 0.61].

Regarding gender, no significant differences were observed for temporal discounting for money in women (*M* = 0.59, SD = 0.25) compared with men (*M* = 0.61, SD = 0.28), *t*(142) = 0.50, *p* = 0.62. Analysis also demonstrated no significant differences in temporal discounting for Bitcoin in women (*M* = 0.60, SD = 0.25) compared with men (*M* = 0.57, SD = 0.34), *t*(142) = 0.60, *p* = 0.55. Note however that significant differences were observed regarding the gender distribution in our sample [χ^2^(1, *N* = 144) = 149.61, *p* < 0.001].

### 3.3. Significant correlations between temporal discounting and day trading

**FIGURE 2:**
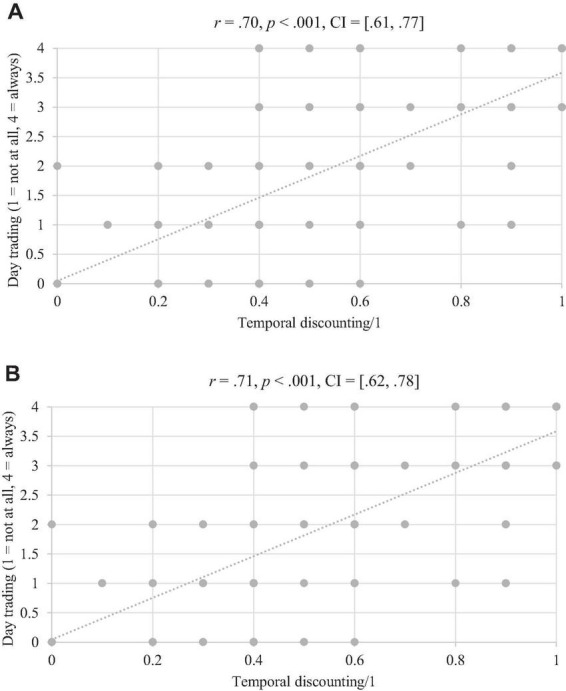
Correlations between day trading and temporal discounting for money **(A)** and Bitcoin **(B)**.

No significant correlations were observed between participants’ age and temporal discounting for money (*r* = 0.021, *p* = 0.80, CI = [−0.14, 0.18]), or for Bitcoin (*r* = 0.019, *p* = 0.84, CI = [−0.14, 0.18]). No significant correlations were observed between participants’ education level and temporal discounting for money (*r* = 0.041, *p* = 0.78, CI = [−0.12, 0.20]), or for Bitcoin (*r* = 0.048, *p* = 0.76, CI = [−0.11, 0.21]). However, and as highlighted in Figure 2, significant positive correlations were observed between day trading and temporal discounting for money and Bitcoin. In other words, the more day trading was reported, the more preferences for short-term rewards were observed. Note that the mean of day trading as reported by the participants was 2.2 (SD = 1.40), near the level (i.e., sometimes) on the day trading item. In addition, significant positive correlations were observed between temporal discounting for money and temporal discounting for Bitcoin (*r* = 0.67, *p* < 0.001, CI = [0.57, 0.75]).

## 4. Discussion

We investigated temporal discounting for money and Bitcoin in short- and long-term investors, this by inviting Bitcoin holders to answer temporal discounting questionnaires dealing with money and Bitcoin. Although we expected higher temporal discounting for Bitcoin than for money in Bitcoin holders, analysis has demonstrated no significant differences between temporal discounting for money and Bitcoin. Thus, Bitcoin holders have shown similar temporal discounting for both money and Bitcoin, although the temporal discounting for both money and Bitcoin were higher than the mean. Critically, analyses have demonstrated higher temporal discounting for both money and Bitcoin in short-term investors compared with long-term investors. In a similar vein, analyses have demonstrated significant positive correlations between day trading and temporal discounting for both money and Bitcoin. These findings are very relevant as they demonstrate how short-term time horizons in Bitcoin holders can be associated with preferences for immediate, over delayed, but larger rewards.

As demonstrated by our analysis, Bitcoin holders tend to demonstrate similar temporal discounting for both money and Bitcoin. In other words, Bitcoin holders seem to prefer immediate over long-term, but higher, money and crypto rewards. Critically, this tendency is associated with time horizons of investment as temporal discounting was larger in short-term rather than in long-term Bitcoin holders. Temporal discounting in Bitcoin holders was also associated with an increase in day trading. Cryptocurrency day trading can be compared with risky investment because the cryptocurrency market is much volatile compared with most traditional stocks. Prices for a given cryptocurrency can increase over 100% and then drop back down to, and even below, the opening price in 1 day (Meng and Fu, 2020). This volatility can even be observed for Bitcoin. Thus, and compared with traditional markets, cryptocurrency trading involves higher uncertainties, especially that the total cryptocurrency market cap fluctuates daily and that many new coins are daily created, with some coins quickly “pumped” and “dumped” (i.e., artificially inflated than deflated in value). Although these extreme financial movements can create some trading opportunities, risks of are high. These day trading decisions can be shortsighted and involve the “rush” experience whereby traditional traders process volatile stocks for the volatility experience *per se* rather than for strategic investment (Arthur et al., 2016b; Youn et al., 2016; Grall-Bronnec et al., 2017). This shortsighted experience may explain why day crypto traders may prefer short-term rewards over long-term, but higher, rewards.

We, here, suggest that short time investment horizons in Bitcoin holders can be associated with shortsighted decisions. Support for this assumption is found in research demonstrating how day trading in general may be somewhat akin to gambling (Granero et al., 2012; Dixon et al., 2018). For instance, day traders tend to engage in gambling activities such as race and sport betting and poker (Arthur and Delfabbro, 2016). Day traders and gamblers also tend to share common sociodemographic features, such as the likelihood of being male, younger and more educated compared with long-term investors (Arthur and Delfabbro, 2016). Further, like gambling, few day traders achieve long-term profits and many day traders tend to be beginners and quickly leave the markets (Jordan and Diltz, 2003). Thus, the increased temporal discounting in day traders in crypto markets may be attributed to their little experience as newcomers so their decision-making is based on limited information and/or technical rather than fundamental analyses.

The increased temporal discounting in short-term Bitcoin traders can be further attributed to several psychological factors as drawn by Delfabbro et al. (2021) to understand the psychology of cryptocurrency traders. One of these factors is the illusion of control, that is, the subjective over-estimation of the ability to exert control on trading (Wohl and Enzle, 2002). This illusion, which is a common feature of gambling, may result in a sense of invincibility in cryptocurrency traders and/or in the false belief that they cannot lose, which contributes to greater risk taking and, consequently, to high temporal discounting by which day traders focus on the “here and now” gains rather than on the long-term rewards which should be typically based on long-term strategies.

Another gambling factor that may explain the increased temporal discounting in cryptocurrency day traders may be preoccupation. Preoccupation is a gambling/addiction feature by which gamblers continuously think about the activity and find it difficult to disengage from it (Griffiths, 2005). Cryptocurrency day trading can be particularly absorbing as traders have continuous and unlimited access to price movements and online media about the cryptocurrency news. Cryptocurrency day traders can also make regular buy and sell decisions on any time per day. This may result in the possibility for cryptocurrency day traders to dedicate a considerable amount of time and attentional resources for trading, resulting in the “here and now” (i.e., temporal discounting) decisions. Beside the illusion of control and preoccupation factors, fear of missing out may also play a role in the increased temporal discounting in cryptocurrency day traders. Fear of missing out refers to the general feelings of anxiety that arise out of the belief that one is missing out on rewarding experiences that others have (Przybylski et al., 2013). In the context of the current study, fear of missing out refers to fear of missing potential gains in the crypto market. When prices of Bitcoin go up, day traders may fear missing out this opportunity (i.e., they may regret not buying before the uptrend). Accordingly, instead of taking profits, they tend to buy the high prices although the coin can be exposed to a subsequent correction. Thus, fear of missing out may result in the “here and now” decisions rather than in the long-term benefited ones. Taken together, the increased temporal discounting in crypto day traders, as observed in our study, can be attributed to several trading-related psychological factors such as the illusion of control, preoccupation, and fear of missing out.

Regarding the demographic data, our analysis did not show associations between temporal discounting and age, education level, or gender. This is different from research showing how decreased risky decision making can increase with age (Mok and Hraba, 1991; Welte et al., 2001). It is worth noting that our sample predominantly consisted of young participants, with an average age of 26.18 years. Although the inclusion of only young participants may be considered as a limitation of our study, cryptocurrencies are relatively new and tend to attract a younger demographic. The same precaution should be applied for education level and gender as our sample size included participants with high education level (*M* formal education = 14.78 years) and were mostly men. This sociodemographic data mirrors however the typical sociodemographic characteristics of cryptocurrency holders. For instance, participants in a study on cryptocurrency trading by Sonkurt and Altinoz (2021) were mostly men and holding university and postgraduate education.

Future research, especially in neuroeconomics, can assess the neural basis of temporal discounting for cryptocurrencies. Previous research in this area has extensively assessed the neural basis of temporal discounting to shed light on the involvement of brain regions such as the ventral striatum, ventromedial prefrontal cortex, posterior cingulate cortex, and the lateral prefrontal cortex (Scheres et al., 2013). In future studies, it would be valuable to investigate whether these brain regions are also involved in temporal discounting for cryptocurrencies and, importantly, whether their activation patterns differ based on the subjective value of immediate and delayed crypto rewards. Additionally, it would be interesting to explore which of these brain regions are more prominently associated with the ability to control the tempting immediate cryptocurrency rewards, particularly in individuals with high levels of impulsivity.

## 5. Conclusion

To summarize, cryptocurrency investment is a rapidly growing activity and is likely to continuously increase and attract more people, especially with the development of applications and platforms that offer fast, continuous, and easy entry into the cryptocurrency world. This world does not only include holding, but also several options of trading, including risky ones. While there is burgeoning research on the characteristics and risks of cryptocurrency trading, the increasing adoption of online applications and platforms of trading should attract more empirical research to better understand the psychological determinants and consequences of this day trading vs. long-term investment.

## Data availability statement

The raw data supporting the conclusions of this article will be made available by the authors, without undue reservation.

## Ethics statement

The studies involving human participants were reviewed and approved by the CERNI. The patients/participants provided their written informed consent to participate in this study.

## Author contributions

ME designed the study. AM contributed to data analysis and interpretation. ME and AM contributed to writing and editing the manuscript. Both authors contributed to the article and approved the submitted version.

## Appendix

The temporal discounting questionnaire

– “Which do you prefer, you get 0.1 Bitcoin right now or 1 Bitcoin in a month?”
– “Which do you prefer, you get 0.2 Bitcoin right now or 1 Bitcoin in a month?”
– “Which do you prefer, you get 0.3 Bitcoin right now or 1 Bitcoin in a month?”
– “Which do you prefer, you get 0.4 Bitcoin right now or 1 Bitcoin in a month?”
– “Which do you prefer, you get 0.5 Bitcoin right now or 1 Bitcoin in a month?”
– “Which do you prefer, you get 0.6 Bitcoin right now or 1 Bitcoin in a month?”
– “Which do you prefer, you get 0.7 Bitcoin right now or 1 Bitcoin in a month?”
– “Which do you prefer, you get 0.8 Bitcoin right now or 1 Bitcoin in a month?”
– “Which do you prefer, you get 0.9 Bitcoin right now or 1 Bitcoin in a month?”
– “Which do you prefer, you get 1 Bitcoin right now or 1 Bitcoin in a month?”
